# NRG1β-overexpressing mesenchymal stem cell-derived exosomes alleviate oxygen-glucose deprivation-mediated neuronal injury via the miR-296-3p/MAOA axis

**DOI:** 10.3389/fncel.2026.1809758

**Published:** 2026-07-27

**Authors:** Kuihua Wang, Haizhen Xu, Xiaohui Ji, Shengjie Zhu, YanJun Lin, Hua Chen, Xiangping Chen, Xuyang Jiang, He Zhou, Jianxin Ye, Xiaoping Cui

**Affiliations:** 1 Fuzong Clinical Medical College of Fujian Medical University, Fuzhou, Fujian, China; 2 900th Hospital of PLA Joint Logistic Support Force, Fuzhou, Fujian, China; 3 Fuzong Teaching Hospital of Fujian University of Traditional Chinese Medicine (900th Hospital), Fuzhou, Fujian, China

**Keywords:** exosomes, MCAO, miR-296-3p, MSC, NRG1β

## Abstract

**Background:**

Cerebral ischemic injury is a severe neurological disorder necessitating effective therapeutic strategies. Neuregulin-1β (NRG1β) and microRNAs are critical for neuroprotection, but the mechanisms by which NRG1β regulates microRNAs in neuronal injury are not fully understood.

**Methods:**

We characterized mesenchymal stem cells (MSCs) through their phenotypic markers and multilineage differentiation potential, confirming NRG1β overexpression in engineered MSCs. Transmission electron microscopy and nanoparticle tracking analysis were used to isolate and characterize exosomes. *In vitro* studies evaluated the effects of MSC/NRG1β-exosomes on OGD-treated HT-22 neuronal cells.

**Results:**

Mesenchymal stem cells/NRG1β-exosomes significantly enhanced HT-22 neuronal survival while reducing apoptosis and reactive oxygen species (ROS). The treatment reduced oxidative stress by lowering malondialdehyde (MDA) levels and boosting superoxide dismutase (SOD) activity. It also inhibited inflammatory cytokines such as IL-1β, TNF-α, and IL-6. Studies conducted *in vivo* with a middle cerebral artery occlusion model demonstrated that MSC/NRG1β-exosomes led to a reduction in infarct size, enhanced histopathological results 24 h after injury. Mechanistically, these exosomes were enriched with miR-296-3p, targeting monoamine oxidase A (MAOA) for downregulation. Inhibition of miR-296-3p or MAOA overexpression negated the protective effects of MSC/NRG1β-exosomes.

**Conclusion:**

Our findings demonstrate that exosomes derived from NRG1β-overexpressing mesenchymal stem cells (MSCs) exert neuroprotective effects against ischemic injury by upregulating miR-296-3p. This microRNA targets MAOA, leading to a reduction in apoptosis, oxidative stress, and inflammation. These results highlight the therapeutic potential of MSC/NRG1β-exosomes and the miR-296-3p/MAOA signaling axis in the treatment of ischemic brain injury.

## Introduction

Cerebral ischemic stroke is one of the leading causes of mortality and long-term disability globally, arising from impeded cerebral blood flow that triggers neuronal damage, inflammatory responses, and oxidative stress (Chen et al., 2021; Maida et al., 2020). Current treatments, such as thrombolytic therapy and mechanical thrombectomy, are limited by narrow therapeutic time windows and variable efficacy, leaving many patients with irreversible neurological deficits (Nakamura et al., 2023; Taha et al., 2025; Zhang et al., 2025). Consequently, the emergence of novel and more effective therapeutic methods to alleviate ischemic brain injury is now a pressing clinical necessity.

Mesenchymal stem cells (MSCs), a promising tool in regenerative medicine, have attracted considerable interest in the field of tissue repair due to their multipotent differentiation capacity, immunomodulatory effects, and paracrine signaling (Caplan, 2017; Han et al., 2019; Wang J. et al., 2020). Evidence suggests that a significant portion of MSCs’ therapeutic benefits stems from their secreted exosomes-membrane-bound vesicles, 30–150 nm in diameter, that transport bioactive molecules including proteins, lipids, and nucleic acids (Hade et al., 2021; Keshtkar et al., 2018). Within these exosomes, microRNAs (miRNAs) serve as critical mediators of neuroprotection by modulating post-transcriptional gene expression (Mendt et al., 2019; Tan et al., 2024). For instance, miRNAs from MSC exosomes have demonstrated the ability to boost neurogenesis, angiogenesis, and synaptic plasticity while reducing neuroinflammation, making them a potential treatment for ischemic stroke (Wei et al., 2022; Zhang et al., 2022). Accordingly, MSC-derived exosomes are increasingly recognized as a valuable neuroprotective tool in cerebral ischemia management.

Neuregulin-1β (NRG1β), a member of the epidermal growth factor family, plays an essential role in neuronal development, synaptic regulation, and neuroprotection (Hao et al., 2014). Studies have revealed that NRG1β confers protective effects in cerebral ischemia models by activating the ErbB receptor signaling pathway, which reduces apoptosis and inflammation (Ennequin et al., 2015; Kuramochi et al., 2004). However, the direct administration of NRG1β protein is hampered by challenges such as rapid degradation, limited blood-brain barrier (BBB) permeability, and off-target effects (Jalilzad et al., 2019). In contrast, employing MSC-derived exosomes as carriers for NRG1β enhances its stability and facilitates targeted delivery to the brain, effectively addressing these shortcomings.

In recent years, genetic engineering of MSCs has unlocked new possibilities for enhancing their therapeutic potential (Shams et al., 2023; Wei et al., 2018). By manipulating the expression of specific genes in MSCs, the bioactive contents of their exosomes can be optimized to bolster their reparative effects. For instance, MSCs engineered to overexpress various neuroprotective factors have exhibited improved outcomes in models of neurological disorders (Zhou et al., 2022). This strategy combines the advantages of MSC-derived exosomes as natural delivery vehicles with the therapeutic benefits of specific factors like NRG1β. The mechanisms by which MSC-derived exosomes exert their neuroprotective effects involve multiple pathways, including the transfer of microRNAs (miRNAs) that regulate gene expression in recipient cells (Nitta et al., 2020). MiRNAs can modulate various cellular processes relevant to ischemic injury, such as apoptosis, inflammation, and oxidative stress (Chen et al., 2020; Hsu et al., 2019). However, the specific miRNAs involved in the protective effects of NRG1β-overexpressing MSC-derived exosomes and their downstream targets remain to be elucidated.

In this study, we investigated the neuroprotective effects of exosomes derived from NRG1β-overexpressing MSCs (MSC/NRG1β-Exo) against oxygen-glucose deprivation (OGD)-induced neuronal injury *in vitro* and middle cerebral artery occlusion (MCAO)-induced brain injury *in vivo*. Furthermore, we explored the molecular mechanisms underlying these protective effects, focusing on the miR-296-3p/MAOA axis. Our findings provide novel insights into the therapeutic potential of genetically engineered MSC-derived exosomes and their molecular mechanisms in ischemic brain injury.

## Materials and methods

### Cell culture and NRG1β overexpression

Mouse bone marrow-derived mesenchymal stem cells (MSCs) were obtained from Thermo Fisher Scientific Inc (Thermo Fisher Scientific, Shanghai, China). The HT-22 mouse hippocampal cell line (IM-M038) was obtained from Immocell (Xiamen, Fujian). Cells were expanded in DMEM/F12 medium supplemented with 10% fetal bovine serum (FBS) and 1% penicillin-streptomycin in a 37°C incubator with 5% CO2. MSCs between passage 3 and 5 were characterized via flow cytometry for surface antigens (CD29, CD44, CD90, CD34, and CD45) and by their capacity to differentiate into osteogenic and adipogenic lineages.

To generate cells overexpressing neuregulin-1β (NRG1β), MSCs were infected with a lentiviral vector encoding the full-length mouse NRG1β (NRG1β). Cells transduced with an empty vector (Vector) served as controls. Stable clones were chosen by treating with 2 μ g/mL puromycin (Sigma Aldrich, MO, USA) for a duration of 14 days. The HT-22 murine hippocampal neuronal cell line was cultured in DMEM with 10% FBS and 1% penicillin-streptomycin under identical incubation conditions.

### Isolation and characterization of MSC-derived exosomes

Mesenchymal stem cells were cultured to approximately 80% confluence and then switched to exosome-depleted medium (DMEM/F12 containing 10% FBS pre-cleared of exosomes) for an additional 48 h. Conditioned medium was collected and exosomes were concentrated using an exosome isolation kit (Shanghai Sangon, Shanghai, China). The isolation protocol was followed according to the manufacturer’s instructions. Briefly, the conditioned medium was subjected to sequential centrifugation at 300 × *g* for 10 min, 2,000 × *g* for 20 min, and 10,000 × *g* for 30 min (4°C) to remove cells and debris. The supernatant was handled with the kit, and exosomes were separated by ultracentrifugation at 100,000 × *g* for 70 min. The exosome pellet was washed with PBS and re-pelleted at 100,000 × g for a further 70 min. The concluding exosome pellet was resuspended in PBS and stored at−80°C for further use.

Exosomes were examined under a transmission electron microscope (TEM; JEOL JEM-1400) following fixation in 2% paraformaldehyde, deposition on carbon-coated copper grids, and negative staining with 2% uranyl acetate. Size distribution was assessed using nanoparticle tracking analysis (NTA) with a NanoSight NS300 (Malvern Instruments). Western blot analysis for CD9, CD81, TSG101 (positive markers), and Calnexin (negative marker) was used to confirm exosome identity.

### The oxygen and glucose deprivation (OGD) experimental model

To simulate ischemic conditions, HT-22 cells were transferred into glucose-free DMEM and placed in a modular hypoxic chamber (1% O2, 5% CO2, 94% N2) for 4 h. Following OGD, cells were returned to normoxic conditions in complete medium. For exosome-treated groups, 100 μ g/mL of MSC-derived exosomes (MSC-Exo or MSC/NRG1β-Exo) was added immediately after OGD. Control cells remained in normal DMEM with 10% FBS under normoxia.

### Middle cerebral artery obstruction (MCAO) model

All animal experiments were approved by the Animal Ethics Committee of the 900th Hospital of the Joint Logistics Support Force (Ethics Approval No. 2023-21) on May 30, 2023. The study, entitled “NRG1β-overexpressing mesenchymal stem cell-derived exosomes alleviate oxygen-glucose deprivation-mediated neuronal injury via the miR-296-3p/MAOA axis,” was conducted in compliance with the institutional guidelines. The reporting of this work is in line with the ARRIVE guidelines 2.0. All procedures adhered to the Guide for the Care and Use of Laboratory Animals (National Research Council, 2011) and the AVMA Guidelines for the Euthanasia of Animals (2020) to ensure humane treatment.

In accordance with institutional animal care and use policies, male C57BL/6 mice (8–10 weeks old, weighing 22–25 g) were subjected to transient middle cerebral artery occlusion (MCAO) as previously described. Mice were anesthetized with 2%–3% isoflurane, and the right common carotid artery was exposed through a midline neck incision. A silicone-coated monofilament (Doccol Corporation, Sharon, MA, USA) was inserted into the internal carotid artery and advanced to occlude the origin of the middle cerebral artery. After 60 min of occlusion, the filament was withdrawn to allow reperfusion.

Mice were randomly assigned to five groups (*n* = 10 per group): (1) Sham (surgery without MCAO); (2) MCAO; (3) MCAO + Saline; (4) MCAO + MSC-Exo; and (5) MCAO + MSC/NRG1β-Exo. Immediately following reperfusion, mice received a tail vein injection of saline or 100 μ g of exosomes resuspended in 200 μ L of PBS. Animals were euthanized 24 h post-reperfusion using an overdose of sodium pentobarbital (100 mg/kg, intraperitoneal injection) followed by cervical dislocation, ensuring rapid and humane termination in accordance with ethical guidelines. Brain tissues and serum samples were collected for subsequent histological, immunological, and biochemical analyses.

For *in vivo* biodistribution tracking, exosomes were labeled with the near-infrared fluorescent dye DiR (1,1′-dioctadecyl-3,3,3′,3′-tetramethylindotricarbocyanine iodide; Thermo Fisher Scientific) according to the manufacturer’s instructions. Briefly, exosomes (100 μ g) were incubated with DiR dye (1 μ M) for 30 min at 37°C, followed by ultracentrifugation at 100,000 × *g* for 70 min to remove unincorporated dye. The labeled exosomes were resuspended in 200 μ L PBS and administered via tail vein injection at the onset of reperfusion. PBS containing free DiR dye alone served as a negative control to exclude non-specific fluorescence signals. Twenty-four hours post-injection, mice were sacrificed and brains were harvested for *ex vivo* fluorescence imaging using an *in vivo* imaging system (IVIS Spectrum, PerkinElmer, MA, USA). Fluorescence signals were quantified using Living Image software (PerkinElmer).

### Tests for cell survival and programmed cell death

To determine cell viability, the Cell Counting Kit-8 assay from Dojindo (Kumamoto, Japan) was employed. HT-22 cells were seeded at a density of 1 × 10^∧^4 cells per well in 96-well plates, exposed to OGD (with or without exosome treatment), and incubated for the specified recovery period. Afterward, 10 μ L of CCK-8 solution (5 mg/mL) was introduced to each well, and the cells were kept at 37°C for 2 h. Absorbance was then read at 450 nm with a microplate reader from BioTek Instruments, VT, USA.

Flow cytometry (BD FACSCalibur) was used to assess cell apoptosis with an Annexin V-FITC/Propidium Iodide (PI) kit, following the instructions provided by the manufacturer (BD Pharmingen, CA, USA). Cells were collected, washed, and resuspended in binding buffer, then stained with Annexin V-FITC and PI before analysis.

### PKH26 labeling and exosome uptake

Exosomes were labeled with the fluorescent dye PKH26 (Sigma-Aldrich) according to the manufacturer’s guidelines. HT-22 cells were incubated with PKH26-labeled exosomes for 24 h. Cells were then fixed with 4% paraformaldehyde, counterstained with DAPI, and imaged via a fluorescence microscope to visualize exosome uptake.

### Measurement of oxidative stress and inflammatory factors

Intracellular reactive oxygen species (ROS) were detected with the fluorescent probe DCFH-DA (10 μ M) (Beyotime, China). Cells were incubated at 37°C for 30 min in the dark, washed in PBS, and observed under a fluorescence microscope. Following the suppliers’ guidelines, malondialdehyde (MDA) levels, MAOA activity, and superoxide dismutase (SOD) activity were assessed using commercial assay kits. The levels of IL-1β, TNF-α, and IL-6 in cell culture media and brain homogenates were measured using ELISA kits from Elabscience Biotechnology Co., Ltd., Wuhan, China, following the manufacturers’ instructions.

### Infarct volume assessment

At 24 h post-MCAO, mice were sacrificed, and brains were quickly removed. Brain slices, 2 mm thick and cut coronally, were incubated in a 2% solution of 2,3,5-triphenyltetrazolium chloride (TTC) at 37°C for 20 min before being fixed in 4% paraformaldehyde. TTC-stained section images were taken, and infarct volumes were measured with ImageJ software.

### Histological and immunofluorescence analysis

Brain tissues were fixed in 4% paraformaldehyde, paraffin-embedded, and sectioned (5 μ m thickness). Sections were stained with hematoxylin-eosin (H&E) for pathological evaluation. For immunofluorescence, slides underwent deparaffinization, rehydration, and antigen retrieval, followed by 5% BSA blocking. Primary antibodies against NRG1β, NeuN, Iba1, MAOA, or TUNEL reagents were applied overnight at 4°C, and sections were then incubated with fluorophore-conjugated secondary antibodies. Nuclei were counterstained with DAPI, and fluorescence images were acquired using a confocal microscope (Leica TCS SP8).

### Enzyme-linked immunosorbent assay (ELISA)

To verify the secretory levels of NRG1β after lentiviral transduction, the concentrations of NRG1β in the MSC culture supernatants were quantified using a Mouse Neuregulin 1 isoform HRG-beta 1 ELISA Kit (Catalog # MBS2605733; MyBioSource, San Diego, CA, USA).

### Quantitative real-time PCR (qRT-PCR)

TRIzol reagent (Thermo Fisher Scientific) was used to extract total RNA, and miRNAs were subsequently purified with a miRNeasy Mini Kit (Qiagen, Germany). cDNA synthesis for miRNAs employed the TaqMan MicroRNA Reverse Transcription Kit. The qRT-PCR amplification was performed using either SYBR Green Master Mix or TaqMan Universal PCR Master Mix (Thermo Fisher Scientific) on a real-time PCR system from Applied Biosystems. U6 snRNA served as the internal control for miRNAs, and GAPDH was used as a control for mRNAs. Relative gene expression levels were determined via the ^∧–ΔΔ*Ct*^ method.

### Western blot analysis

Cells and tissues were lysed in RIPA buffer (Sigma Aldrich, MO, USA) containing protease inhibitors (Sigma Aldrich), and protein concentrations were determined using the BCA assay (Thermo Fisher Scientific). Equal amounts of protein (30 μ g) were separated on SDS-PAGE and transferred to PVDF membranes. Membranes were blocked with 5% non-fat milk and probed overnight at 4°C with primary antibodies: NRG1β (isoform SMDF, Polyclonal antibody, 10527-1-AP, Wuhan SanYing), CD9 (Monoclonal antibody, Cat No. 60232-1-Ig, Wuhan SanYing), TSG101 (Monoclonal antibody, Cat No. 67381-1-Ig, Wuhan SanYing), Calnexin (Monoclonal antibody, Cat No. 66903-1-Ig, Wuhan SanYing), CD81 (Polyclonal antibody, 27855-1-AP, Wuhan SanYing), Bax (D2E11) Rabbit mAb #5023, Bcl-2 (D55G8) Rabbit mAb #4223, and MAOA (Antibody #66935) from Cell Signaling Technology (CST, MA, USA). After incubation with horseradish peroxidase (HRP)-conjugated secondary antibodies, immunoreactive bands were visualized using an enhanced chemiluminescence reagent. Densitometry of the bands was performed using ImageJ software.

### Reporter assay with dual-luciferase

The 3′ untranslated region (3′UTR) of MAOA containing the predicted miR-296-3p binding motif was cloned into a pMIR-REPORT miRNA Expression Reporter Vector (MAOA-WT). A mutant version of the 3′UTR (mutated in the miR-296-3p target site) was also generated (MAOA-MUT). HEK293T cells were co-transfected with these plasmids and either miR-296-3p mimic, inhibitor, or corresponding negative controls. After 48 h, luciferase activity was measured using a Dual-Luciferase Reporter Assay Kit (Promega, WI, USA), following standard protocols.

### RNA immunoprecipitation (RIP)

According to the manufacturer’s instructions, RNA immunoprecipitation was carried out using a Magna RIP Kit from Millipore, Merck Millipore Darmstadt, Germany. To summarize, HT-22 cell lysates (following exosome treatment) were exposed to magnetic beads bound to anti-Ago2 or an isotype-matched IgG control. RNA recovered from the beads after proteinase K digestion was analyzed by qRT-PCR to determine whether miR-296-3p and MAOA mRNA were co-precipitated.

### Cell transfection

Using Lipofectamine RNAiMAX, HT-22 cells were transfected with either a miR-296-3p inhibitor (anti-miR-296-3p) or a negative control (anti-NC). For MAOA overexpression, cells were transfected with pCDNA3.1-MAOA or an empty vector using Lipofectamine 3000. Transfection efficiency was verified via qRT-PCR or western blot.

### Statistical analysis

All *in vitro* experiments were independently performed in triplicate (*n* = 3 independent biological replicates), with each biological replicate containing three technical replicates to ensure intra-experimental consistency. For *in vivo* studies, the sample size (*n* = 10 mice per group) was determined by a power analysis based on preliminary data and previous studies with similar experimental designs, aiming for a power of 0.80 and a significance level of 0.05 to detect expected effect sizes. Quantitative data are expressed as mean ± standard deviation (SD). To allow direct visual assessment of sample size and data distribution, individual data points are displayed as dot-bar plots in all figures. All statistical analyses were conducted using GraphPad Prism 8.0. Prior to parametric testing, data normality was verified using the Shapiro-Wilk test, and homogeneity of variance was assessed using the Brown-Forsythe or Levene’s test. For comparisons between two groups, an unpaired Student’s *t*-test was employed. For multiple group comparisons, a one-way ANOVA followed by Tukey’s *post-hoc* test was used. For data that did not follow a normal distribution, the Kruskal-Wallis test followed by Dunn’s *post-hoc* test was applied. A *P*-value < 0.05 was considered statistically significant. Statistical significance is denoted as:**p < 0.01 vs. Normal group; &*p* < 0.05 vs. DN group; #*p* < 0.05 vs. DN + sh-TRIB3 group.

## Results

### Characterization of MSCs and NRG1β-overexpressing MSC-derived exosomes

**FIGURE 1:**
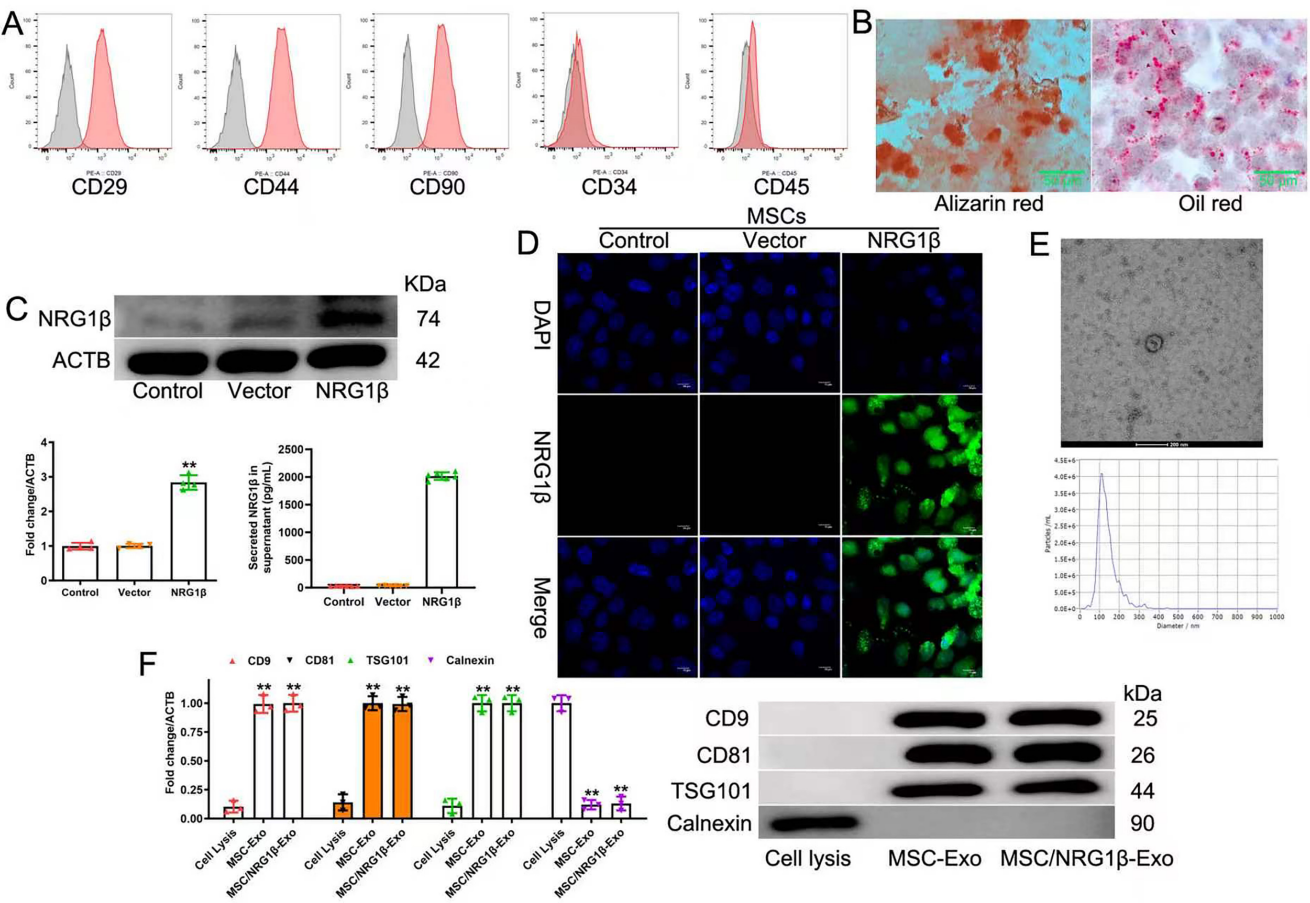
Characterization of MASs and MSC-derived exosomes with NRG1β overexpression. **(A)** Flow cytometry analysis of MSC surface markers (CD29, CD44, CD90, CD34, and CD45). Gray histograms represent the isotype controls, and red histograms represent specific antibody staining. **(B)** Multilineage differentiation of MSCs. Alizarin Red staining (left) and Oil Red staining (right) confirm osteogenic and adipogenic potential, respectively. Scale bar = 50 μ m. **(C)** Validation of NRG1β overexpression. Upper: Representative Western blot. Lower right: ELISA quantification of secreted NRG1β levels in culture supernatants (*n* = 6 biological replicates). **(D)** Representative immunofluorescence images of NRG1β (green) in Control, Vector, and NRG1β-transfected MSCs. Nuclei were counterstained with DAPI (blue). Scale bar = 10 μ m. **(E)** Characterization of exosomes. Left: Transmission electron microscopy (TEM) image showing cup-shaped morphology. Scale bar = 200 nm. Right: Nanoparticle tracking analysis (NTA) showing size distribution. Scale bar = 200 nm **(F)** Western blot analysis of exosomal positive markers (CD9, CD81, TSG101) and negative marker (Calnexin) in exosomes and cell lysates. Data represent *n* = 3 independent experiments. **p < 0.01.

Our initial investigations focused on establishing the identity and properties of the mesenchymal stem cells (MSCs) and their derived exosomes. Flow cytometry analysis demonstrated that the isolated cells exhibited the typical immunophenotypic profile of MSCs, with high expression of positive surface markers CD29 (98.6%), CD44 (98.8%), and CD90 (98.9%), while showing negligible expression of hematopoietic lineage markers CD34 (2.48%) and CD45 (2.70%) (Figure 1A). The multipotency of these MSCs was further validated through differentiation assays. The presence of calcium deposits visualized by Alizarin Red staining and lipid droplet accumulation detected by Oil Red staining confirmed their osteogenic and adipogenic differentiation capacities, respectively (Figure 1B). Having established the stemness properties of our MSCs, we proceeded to generate NRG1β-overexpressing MSCs. Western blot analysis revealed substantial upregulation of NRG1β protein in the genetically modified cells compared to control MSCs and vector-transfected cells, whileβ-actin levels remained consistent across all groups, confirming successful overexpression (Figure 1C, upper panel). To confirm the functional secretion of NRG1β, we measured the levels of NRG1β in cell culture supernatants by ELISA. The results showed that NRG1β-overexpressing MSCs secreted significantly higher levels of NRG1β compared to negligible levels in the control and vector groups (Figure 1C, lower right panel). Furthermore, immunofluorescence staining validated the intracellular expression of NRG1β, showing strong green fluorescence signal specifically in NRG1β-transfected cells, while control and vector groups exhibited minimal background fluorescence (Figure 1D). Transmission electron microscopy revealed the characteristic cup-shaped morphology of the isolated vesicles, with diameters predominantly ranging from 100 to 200 nm (Figure 1E, upper panel). Nanoparticle tracking analysis confirmed a typical exosomal size distribution, with a peak diameter around 100–150 nm (Figure 1E, lower panel). The purity of the exosome preparations was further assessed by calculating the particle-to-protein ratio. The results showed a ratio of approximately 1.2 × 10^∧^10 particles/mg protein, indicating a high degree of purity consistent with the MISEV guidelines. To confirm the purity and identity of these exosomal preparations, we performed Western blot analysis for established exosomal markers. Both control MSC-derived and NRG1β-MSC-derived exosomes showed strong expression of CD9, CD81, and TSG101, while being negative for the endoplasmic reticulum protein calnexin, which was detected only in the cellular lysates (Figure 1F). Notably, the exosomal marker profile remained consistent between control and NRG1β-overexpressing MSC-derived exosomes, indicating that NRG1β overexpression did not alter the fundamental characteristics of the secreted vesicles.

### Exosomes derived from NRG1β-overexpressing MSCs provide neuroprotection in a mouse model of cerebral ischemia

**FIGURE 2:**
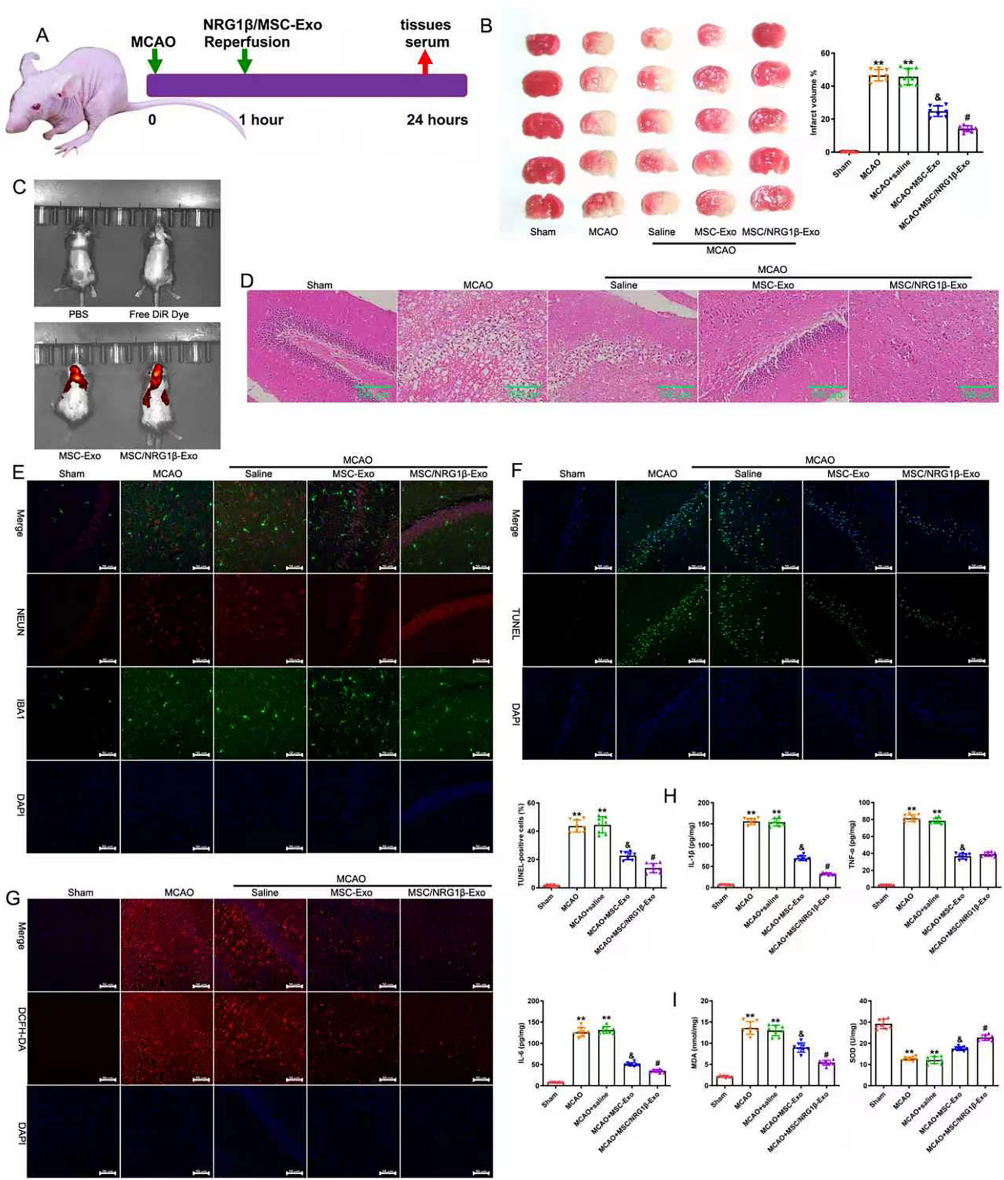
Mesenchymal stem cell/Neuregulin-1β (MSC/NRG1β)-Exo ameliorate brain injury in a MCAO model of stroke. **(A)** Schematic of the experimental timeline. **(B)** Representative images of TTC-stained brain sections from five experimental groups (Sham, MCAO, MCAO + Saline, MCAO + MSC-Exo, and MCAO + MSC/NRG1β-Exo). (*n* = 10 mice per group). **(C)** *In vivo* fluorescence imaging of exosome distribution. Representative fluorescence images of MCAO mice 24 h after systemic administration of PBS, Free DiR dye, MSC-Exo, or MSC/NRG1β-Exo. **(D)** H&E staining of brain sections demonstrating histopathological changes. Scale bar = 100 μ m. **(E)** Immunofluorescence staining for neuronal marker (NeuN, red) and microglial marker (Iba1, green), with DAPI (blue) nuclear counterstaining across all experimental groups. Scale bar = 50 μ m. **(F)** Immunofluorescence staining for TUNEL (green) with DAPI (blue) counterstaining, showing reduced apoptosis in the MSC/NRG1β-Exo group compared to MCAO, MCAO + Saline, and MCAO+MSC-Exo groups. Scale bar = 50 μ m. **(G)** Immunofluorescence staining for oxidative stress marker DCF-DA (red) with DAPI (blue) counterstaining, demonstrating reduced oxidative stress in MSC/NRG1β-Exo-treated mice compared to other MCAO groups. Scale bar = 50 μ m. **(H)** Quantification of neuroinflammatory markers IL-1β, TNF-α, and IL-6 levels across experimental groups. **(I)** Quantification of MDA (lipid peroxidation marker) and SOD (antioxidant enzyme) levels. **p < 0.01 vs. Sham; &*p* < 0.05 vs. MCAO; #*p* < 0.05 vs. MCAO + MSC-Exo.

Following our comprehensive characterization of MSCs and their derived exosomes, we sought to evaluate the therapeutic potential of NRG1β-overexpressing MSC-derived exosomes (MSC/NRG1β-Exo) *in vivo* using a clinically relevant mouse model of cerebral ischemia. We established a middle cerebral artery occlusion (MCAO) model with a defined experimental timeline: MCAO surgery at baseline, followed by reperfusion and exosome administration at 1-h post-occlusion, with subsequent tissue collection and analysis at 24 h post-reperfusion (Figure 2A). To assess the impact of exosome treatment on infarct volume, we performed TTC staining of brain sections across five experimental groups. While the sham group exhibited uniformly viable tissue, the MCAO and saline-treated groups displayed extensive white infarct regions, indicating significant tissue damage. Notably, administration of MSC-derived exosomes reduced infarct volume, with MSC/NRG1β-Exo treatment demonstrating the most pronounced protective effect (Figure 2B). To track the biodistribution and verify the delivery efficiency of the exosomes, *in vivo* fluorescence imaging was performed 24 h post-administration. DiR-labeled MSC-Exo and MSC/NRG1β-Exo both exhibited robust accumulation within the ischemic brain region, confirming successful delivery across the compromised blood-brain barrier (BBB). Importantly, mice injected with PBS or free DiR dye alone showed negligible fluorescence signals in the brain, ruling out false-positive results arising from passive dye diffusion or non-specific leakage through the injured BBB (Figure 2C). This observation was corroborated by H&E staining, which revealed that MSC/NRG1β-Exo treatment substantially preserved neuronal architecture and attenuated cellular loss compared to the other MCAO groups (Figure 2D). Double staining for NeuN (neurons) and IBA1 (microglia) revealed that MSC/NRG1β-Exo treatment significantly enhanced neuronal survival while concurrently suppressing microglial activation, indicating dual neuroprotective and anti-inflammatory effects that attenuated the neuroinflammatory response in the ischemic region (Figure 2E). Importantly, TUNEL staining confirmed that MSC/NRG1β-Exo treatment markedly reduced apoptotic cell death compared to both untreated and MSC-Exo-treated MCAO groups. Quantitative analysis showed that the percentage of TUNEL-positive cells was significantly lower in the MSC/NRG1β-Exo group compared to other MCAO groups (Figure 2F). Furthermore, DCFH-DA staining demonstrated that MSC/NRG1β-Exo treatment markedly reduced reactive oxygen species (ROS) accumulation in the ischemic brain region compared to other MCAO groups (Figure 2G). To elucidate the molecular mechanisms underlying these neuroprotective effects, we quantified inflammatory and oxidative stress markers in brain tissue. MSC/NRG1β-Exo treatment significantly reduced the levels of pro-inflammatory cytokines IL-1β, TNF-α, and IL-6 compared to the MCAO and saline groups, with greater efficacy than conventional MSC-Exo treatment (Figure 2H). Moreover, MSC/NRG1β-Exo administration effectively mitigated oxidative stress, as evidenced by decreased malondialdehyde (MDA) levels and restored superoxide dismutase (SOD) activity (Figure 2I). Collectively, these findings demonstrate that exosomes derived from NRG1β-overexpressing MSCs exert potent neuroprotective effects in cerebral ischemia by preserving neuronal viability, attenuating neuroinflammation, and reducing oxidative stress.

### Exosomes derived from NRG1β-overexpressing MSCs protect against OGD-induced neuronal injury *in vitro*

**FIGURE 3:**
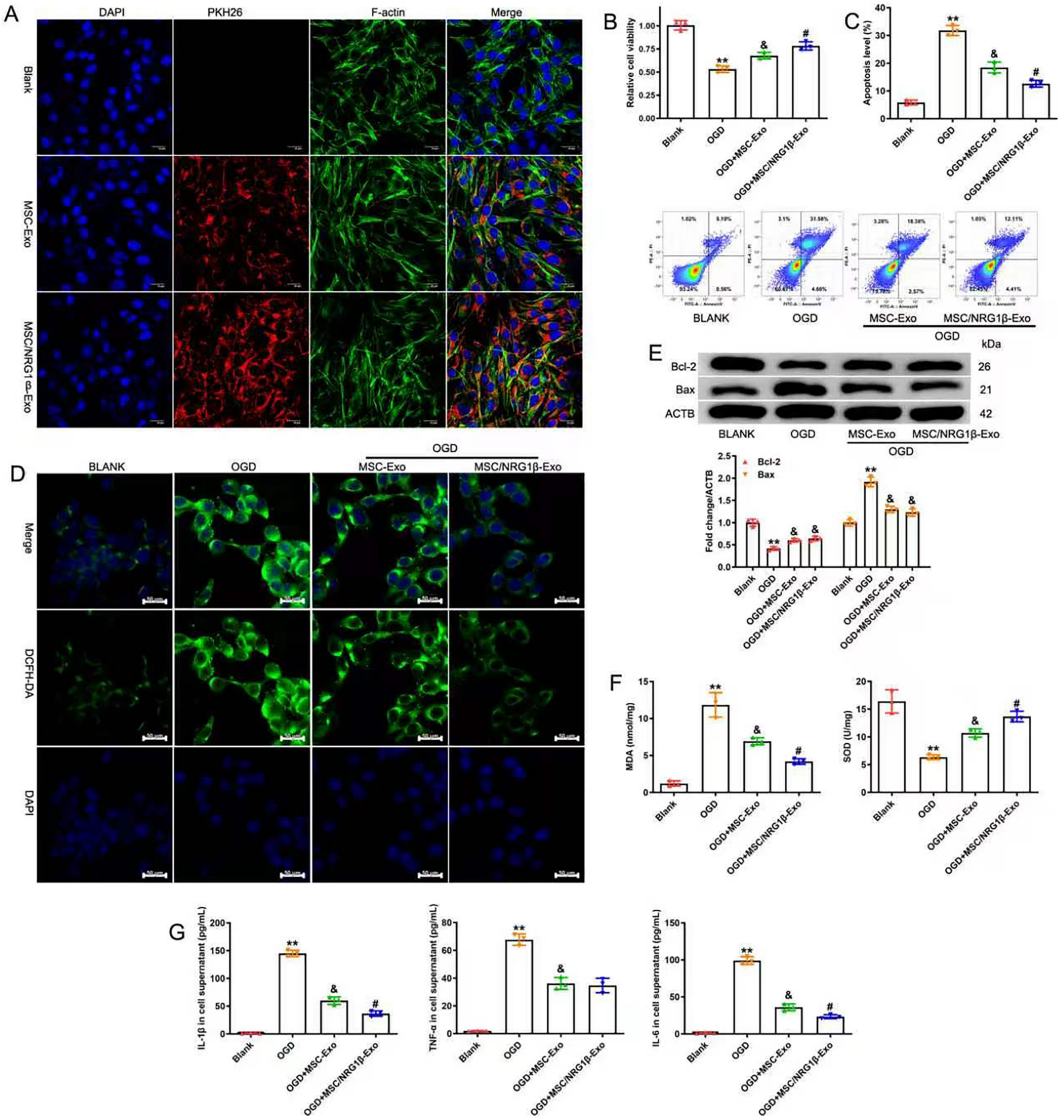
Neuregulin-1β (NRG1β)-overexpressing MSC-derived exosomes protect neuronal cells from oxygen-glucose deprivation (OGD) injury via miR-296-3p enrichment. **(A)** Fluorescence microscopy images of PKH26-labeled exosomes (red) internalized by HT-22 cells. DAPI (blue) counterstaining shows cell nuclei. **(B)** Cell viability assessment using CCK-8 assay. **(C)** Flow cytometry analysis of apoptosis using Annexin V/PI staining. **(D)** DCFH-DA fluorescence staining (green) for reactive oxygen species (ROS) detection with DAPI (blue) counterstaining. Scale bar = 50 μ m. **(E)** Western blot analysis of apoptosis-related proteins Bcl-2 (anti-apoptotic) and Bax (pro-apoptotic). **(F)** Quantification of MDA (lipid peroxidation marker) and SOD (antioxidant enzyme) levels in HT-22 cells. **(G)** ELISA analysis of inflammatory cytokines IL-1β, TNF-α, and IL-6 in cell culture supernatants. All *in vitro* data represent *n* = 3 independent biological experiments. **p < 0.01 vs. Blank; &*p* < 0.05 vs. OGD; #*p* < 0.05 vs. OGD + MSC-Exo.

Having established the neuroprotective effects of MSC/NRG1β-Exo *in vivo*, our next step was to clarify the underlying mechanisms through a series of *in vitro* experiments using HT-22 hippocampal neuronal cells under oxygen-glucose deprivation (OGD) conditions, a widely used model that recapitulates key aspects of ischemic injury. To verify that HT-22 cells could effectively internalize these exosomes, we labeled both MSC-Exo and MSC/NRG1β-Exo with PKH26 dye and tracked their uptake using confocal fluorescence microscopy. Robust cytoplasmic uptake of PKH26-labeled exosomes was observed in HT-22 cells, with F-actin-defined boundaries confirming their intracellular localization (Figure 3A). We next evaluated the functional impact of exosome treatment on HT-22 cells subjected to OGD. Cell viability assays demonstrated that OGD dramatically reduced neuronal survival, while treatment with MSC-Exo partially rescued this effect. Notably, MSC/NRG1β-Exo provided significantly greater protection against OGD-induced cell death compared to MSC-Exo (Figure 3B), mirroring our *in vivo* observations. To assess whether this enhanced cell survival was associated with reduced apoptosis, we performed flow cytometry analysis with Annexin V/PI staining. OGD markedly increased the proportion of apoptotic cells, while MSC/NRG1β-Exo treatment most effectively attenuated this response (Figure 3C). Given that oxidative stress is a critical mediator of neuronal injury following ischemia, we next examined intracellular ROS levels using DCFH-DA staining. OGD exposure markedly increased ROS accumulation, which was partially attenuated by MSC-Exo treatment and most effectively suppressed by MSC/NRG1β-Exo (Figure 3D). To delineate the molecular mechanisms underlying this anti-apoptotic effect, we examined the expression of key regulators of cell death. Western blot analysis revealed that OGD significantly decreased levels of the anti-apoptotic protein Bcl-2 while increasing expression of the pro-apoptotic protein Bax. Treatment with MSC/NRG1β-Exo effectively reversed these changes, promoting a more favorable Bcl-2/Bax ratio compared to MSC-Exo (Figure 3E). Given that oxidative stress and inflammation are critical mediators of neuronal injury following ischemia, we also assessed markers of these processes. MSC/NRG1β-Exo treatment significantly reduced lipid peroxidation (decreased MDA levels) while enhancing antioxidant defense (increased SOD activity) compared to both untreated OGD cells and those receiving MSC-Exo (Figure 3F). Furthermore, examining inflammatory cytokines in cell culture supernatants showed that MSC/NRG1β-Exo was most effective in reducing OGD-induced IL-1β, TNF-α, and IL-6 production (Figure 3G). Collectively, these *in vitro* results support our *in vivo* findings, showing that exosomes from NRG1β-overexpressing MSCs safeguard neuronal cells from OGD-induced damage via various complementary mechanisms.

### Exosomes derived from NRG1β-overexpressing MSCs protect against OGD-induced neuronal injury via miR-296-3p upregulation

**FIGURE 4:**
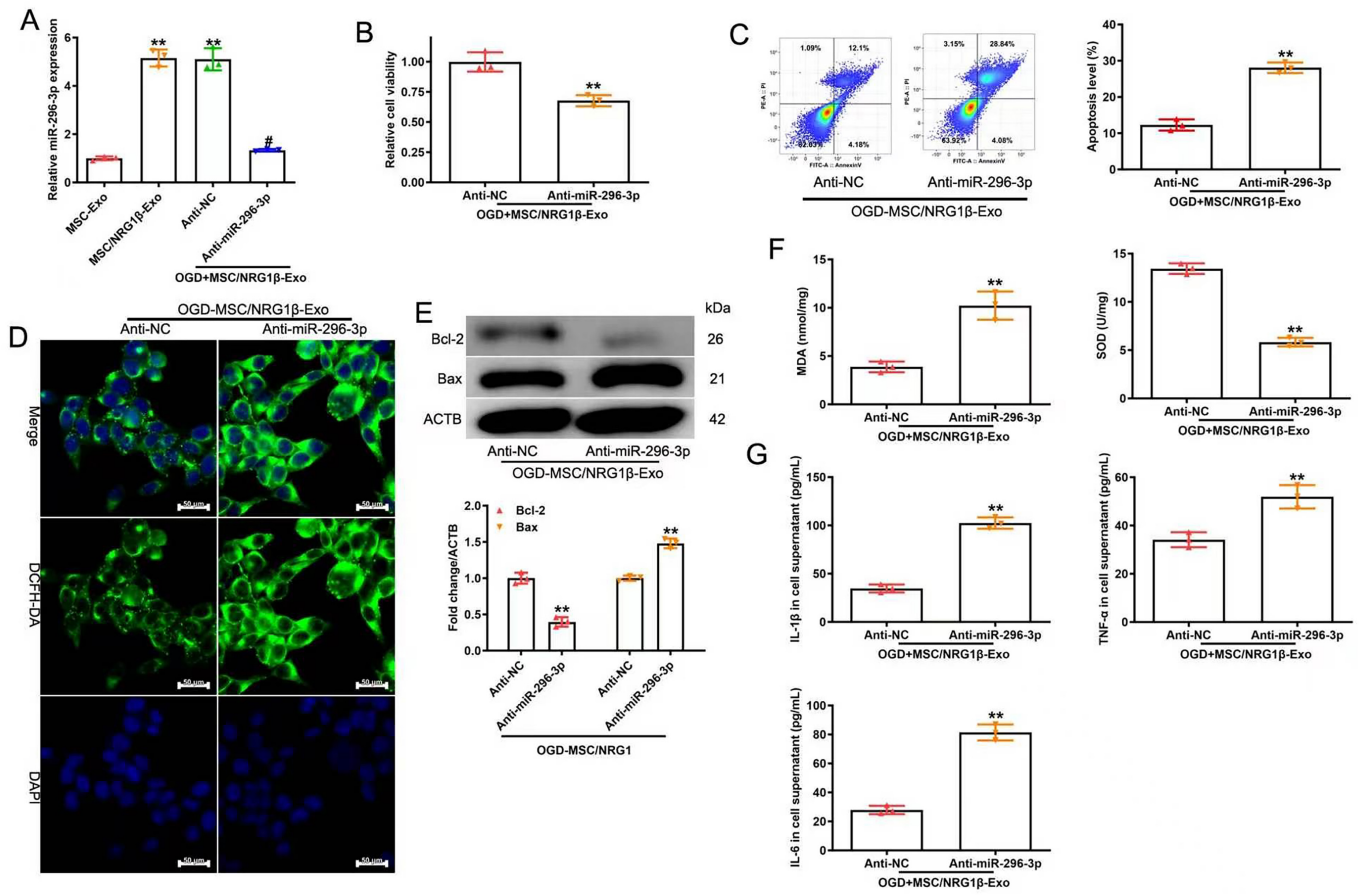
Inhibition of miR-296-3p attenuates the neuroprotective effects of NRG1β-overexpressing MSC-derived exosomes. **(A)** qRT-PCR analysis confirming successful inhibition of miR-296-3p in OGD-injured HT-22 cells treated with MSC/NRG1β-Exo. **(B)** Cell viability assessment by CCK-8 assay showing that inhibition of miR-296-3p significantly reduced the protective effect of MSC/NRG1β-Exo on OGD-injured neuronal cells. **(C)** Flow cytometry analysis of apoptosis using Annexin V/PI staining. **(D)** DCFH-DA fluorescence staining (green) for reactive oxygen species (ROS) detection with DAPI (blue) counterstaining. Scale bar = 50 μ m. **(E)** Western blot analysis of apoptosis-related proteins showing decreased Bcl-2 (anti-apoptotic) and increased Bax (pro-apoptotic) expression in the Anti-miR-296-3p group compared to Anti-NC. **(F)** Quantification of oxidative stress markers in OGD-injured cells treated with MSC/NRG1β-Exo. **(G)** ELISA analysis of inflammatory cytokines in cell culture supernatants. All *in vitro* data represent *n* = 3 independent biological experiments. **p < 0.01 vs. Anti-NC.

Earlier research has indicated that NRG1β is capable of modulating the expression of certain microRNAs, such as miR-296-3p and miR-200c*, which are implicated in cellular protection mechanisms (Sun et al., 2011). In this study, we found through quantitative real-time PCR (qRT-PCR) analysis that miR-296-3p expression was significantly elevated in exosomes derived from NRG1β-overexpressing MSCs (MSC/NRG1β-Exo) compared to control MSC-derived exosomes (Figure 4A). To determine whether the protective effects observed in our previous experiments were dependent on miR-296-3p activity, we performed loss-of-function experiments using a miR-296-3p inhibitor (Anti-miR-296-3p). We first confirmed the efficacy of miR-296-3p inhibition in HT-22 cells treated with MSC/NRG1β-Exo under OGD conditions. Quantitative analysis demonstrated that transfection with the miR-296-3p inhibitor significantly reduced intracellular miR-296-3p levels compared to cells transfected with a negative control inhibitor (Anti-NC) (Figure 4A), cell viability tests showed that reducing miR-296-3p greatly diminished the capacity of MSC/NRG1β-Exo to maintain neuron survival in OGD conditions (Figure 4B). This finding indicates that miR-296-3p is essential for the cytoprotective effects of these exosomes. Consistent with this observation, flow cytometry analysis demonstrated that miR-296-3p inhibition more than doubled the proportion of apoptotic cells despite the presence of MSC/NRG1β-Exo (Figure 4C). The functional importance of miR-296-3p was further illustrated through detection of intracellular ROS using DCFH-DA staining. While MSC/NRG1β-Exo treatment effectively suppressed OGD-induced ROS accumulation in control cells, miR-296-3p inhibition resulted in markedly increased fluorescence intensity, indicating elevated oxidative stress despite exosome treatment (Figure 4D). At the molecular level, Western blot analysis revealed that miR-296-3p inhibition reversed the beneficial effects of MSC/NRG1β-Exo on apoptotic signaling, resulting in decreased expression of the anti-apoptotic protein Bcl-2 and increased levels of the pro-apoptotic protein Bax (Figure 4E). We also investigated whether miR-296-3p mediated the antioxidant and anti-inflammatory effects of MSC/NRG1β-Exo. Inhibition of miR-296-3p significantly increased MDA levels while reducing SOD activity (Figure 4F), indicating compromised antioxidant capacity. Similarly, the suppression of miR-296-3p resulted in increased levels of pro-inflammatory cytokines IL-1β, TNF-α, and IL-6 (Figure 4G), demonstrating that miR-296-3p is essential for the anti-inflammatory properties of MSC/NRG1β-Exo. Collectively, these loss-of-function experiments provide compelling evidence that miR-296-3p serves as a critical mediator of the multifaceted neuroprotective effects of NRG1β-overexpressing MSC-derived exosomes.

### miR-296-3p directly targets MAOA to mediate neuroprotection by NRG1β-overexpressing MSC-derived exosomes

**FIGURE 5:**
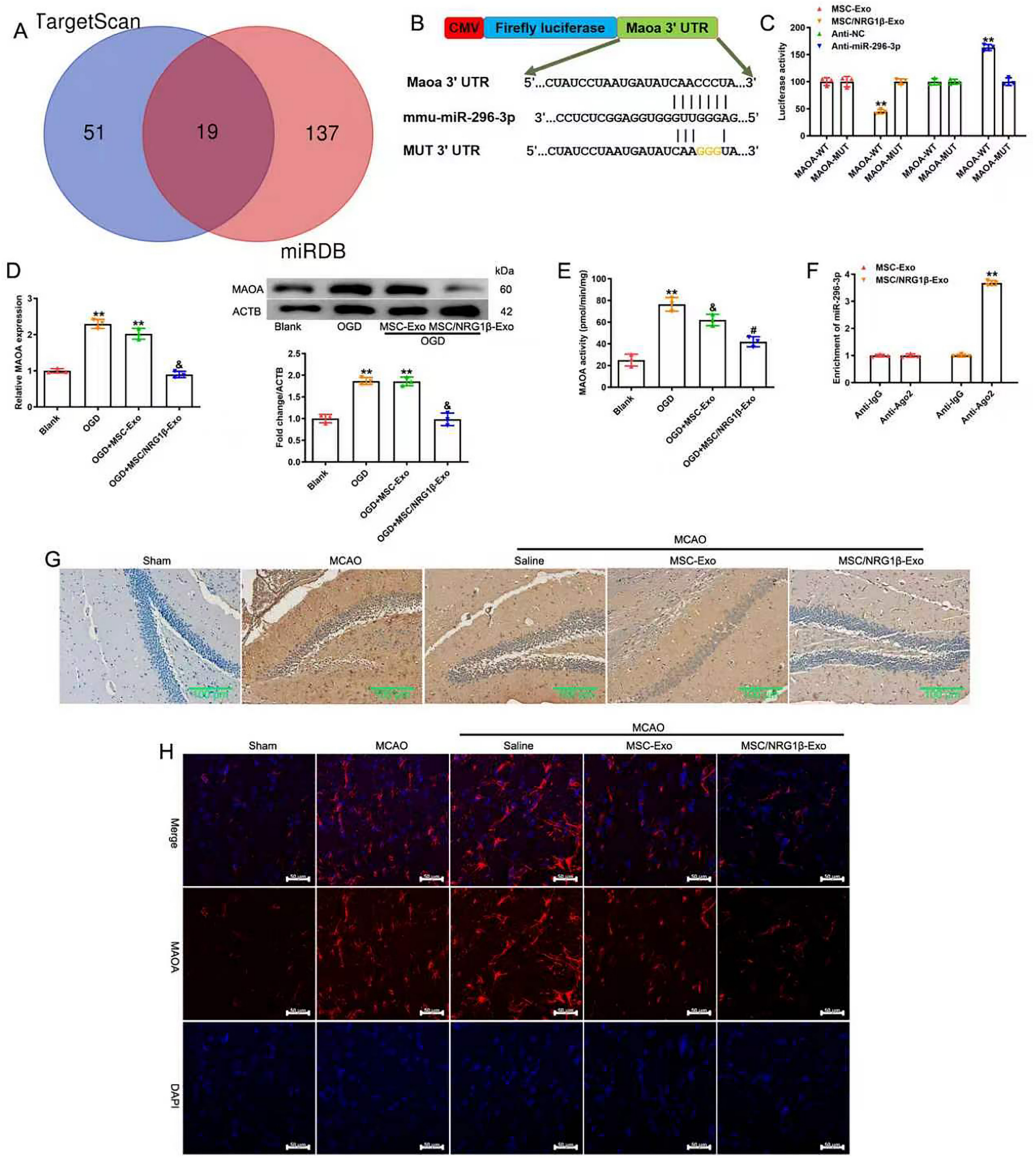
Monoamine oxidase A (MAOA) is a direct target of miR-296-3p in the neuroprotective mechanism of NRG1β-overexpressing MSC-derived exosomes. **(A)** Venn diagram showing the overlap of predicted miR-296-3p target genes between TargetScan and miRDB databases. **(B)** Schematic representation of the luciferase reporter construct containing MAOA 3′ UTR. The binding site sequence between MAOA 3′ UTR and mmu-miR-296-3p is shown with vertical lines indicating complementary base pairing. The mutated nucleotides in the MAOA 3′ UTR binding site (MUT 3′ UTR) are highlighted in yellow. **(C)** Luciferase activity was significantly reduced in wild-type MAOA 3′ UTR when co-transfected with MSC/NRG1β-Exo compared to MSC-Exo, while no significant change was observed with the mutated MAOA 3′ UTR. Anti-miR-296-3p rescued the suppression of luciferase activity. **(D)** qRT-PCR and Western blot analysis assess MAOA expression analysis. **(E)** MAOA enzymatic activity assay showing upregulation after OGD and suppression by MSC/NRG1β-Exo. **(F)** RNA immunoprecipitation (RIP) assay showing significant enrichment of miR-296-3p in Ago2 immunoprecipitates from MSC/NRG1β-Exo-treated cells compared to MSC-Exo-treated cells. **(G)** Immunohistochemical staining of MAOA in brain sections from different experimental groups. Scale bar = 100 μ m. **(H)** Immunofluorescence staining of MAOA (red) with DAPI (blue) nuclear counterstaining in brain sections. Scale bar = 50 μ m. All *in vitro* data represent *n* = 3 independent biological experiments. **p < 0.01. # and & indicate statistically significant differences between the indicated experimental groups.

Having established that miR-296-3p is essential for the neuroprotective effects of MSC/NRG1β-Exo, we next sought to identify the downstream molecular targets through which this microRNA exerts its protective functions. Bioinformatic analysis using complementary prediction algorithms, TargetScan and miRDB, identified 51 and 137 potential target genes for miR-296-3p, respectively. Notably, 19 genes were predicted by both tools, among which monoamine oxidase A (MAOA) emerged as a particularly promising candidate (Figure 5A), given its established role in neuronal injury via promoting oxidative stress and mitochondrial dysfunction. We conducted a dual-luciferase reporter assay with constructs containing either the wild-type or mutated 3′-untranslated region of MAOA to confirm the direct interaction between miR-296-3p and MAOA. The wild-type construct contained the predicted miR-296-3p binding site, while the mutant version harbored a modified sequence designed to disrupt miR-296-3p binding (Figure 5B). Co-transfection with the wild-type MAOA 3′UTR reporter and MSC/NRG1β-Exo significantly reduced luciferase activity compared to MSC-Exo, whereas no change was observed with the mutant reporter; anti-miR-296-3p reversed this suppression (Figure 5C). These results confirm that miR-296-3p directly binds to the MAOA 3’UTR and inhibits its expression. Quantitative analysis revealed that OGD significantly increased MAOA mRNA expression compared to untreated cells, while MSC/NRG1β-Exo treatments reduced MAOA levels, establishing a causal relationship between miR-296-3p and MAOA regulation. Consistent with these findings, Western blot analysis demonstrated that MSC/NRG1β-Exo treatment most effectively reduced MAOA protein levels under OGD conditions (Figure 5D). Importantly, to confirm the functional consequences of MAOA regulation, we measured its enzymatic activity. OGD exposure markedly increased MAOA activity, which was significantly suppressed by MSC/NRG1β-Exo treatment, whereas inhibition of miR-296-3p abolished this suppression (Figure 5E). To further validate this interaction in the context of our exosome treatments, we performed RNA immunoprecipitation (RIP) assays. We observed significantly higher enrichment of miR-296-3p in MAOA mRNA in cells treated with MSC/NRG1β-Exo compared to those treated with control MSC-Exo (Figure 5F), supporting the functional relevance of this miRNA-mRNA interaction in our experimental system. To determine whether this regulatory mechanism operates *in vivo*, we examined MAOA expression in brain tissue from our MCAO model. Immunohistochemical staining revealed minimal MAOA expression in sham-operated animals, with marked upregulation following MCAO. While MSC-Exo treatment modestly reduced MAOA levels, MSC/NRG1β-Exo administration resulted in the most pronounced reduction among all MCAO groups (Figure 5G). These findings were further confirmed by immunofluorescence analysis, which showed a similar pattern of MAOA expression across the experimental groups (Figure 5H). Collectively, these results provide compelling evidence that miR-296-3p directly targets and negatively regulates MAOA, and that this regulatory axis is effectively modulated by MSC/NRG1β-Exo treatment.

### MAOA overexpression counteracts the neuroprotective effects of NRG1β-overexpressing MSC-derived exosomes

**FIGURE 6:**
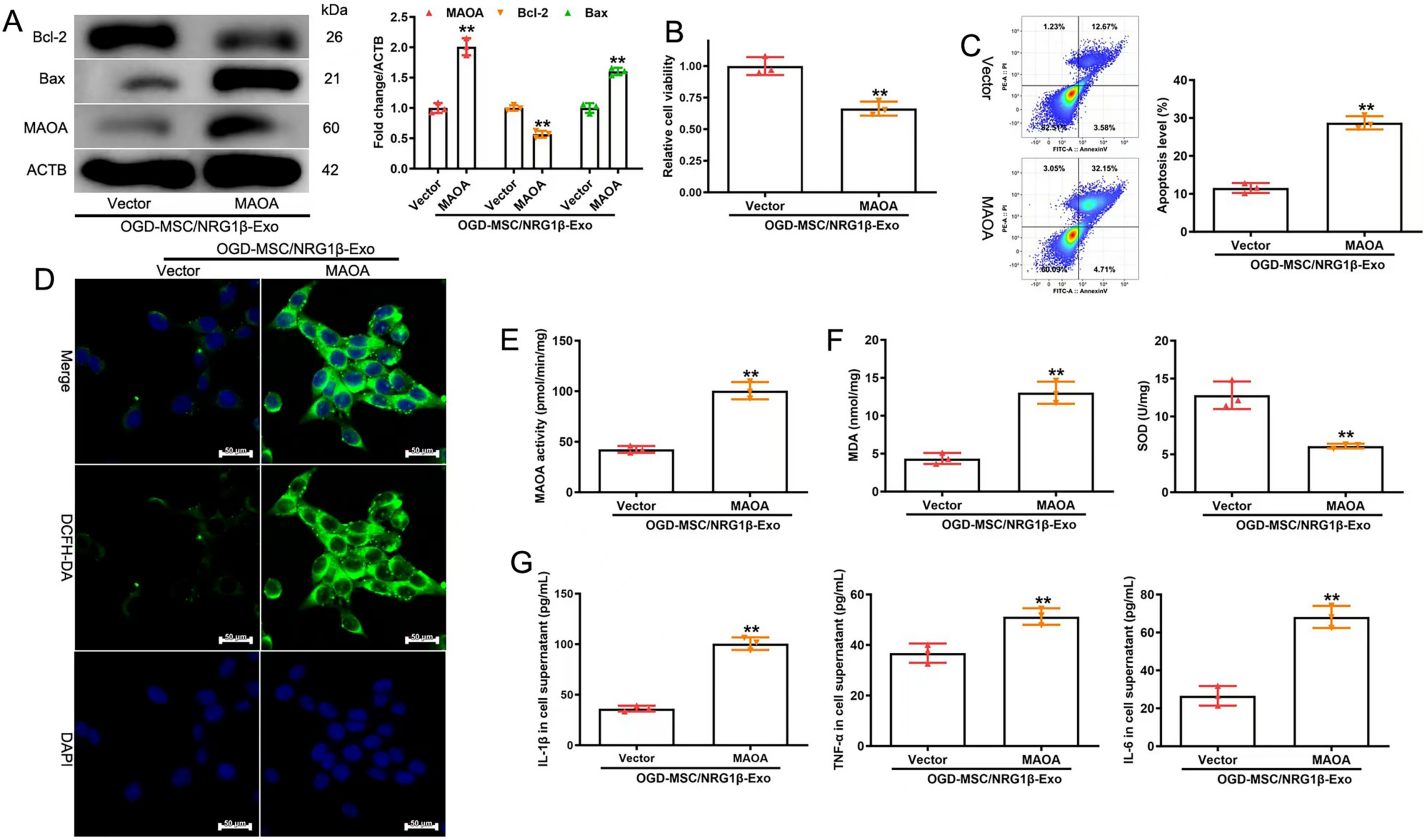
Overexpression of MAOA counteracts the neuroprotective effects of NRG1β-overexpressing MSC-derived exosomes on OGD-injured neuronal cells. **(A)** Western blot analysis confirming successful overexpression of MAOA in HT-22 cells treated with MSC/NRG1β-Exo under OGD conditions. MAOA overexpression decreased anti-apoptotic Bcl-2 expression and increased pro-apoptotic Bax expression. **(B)** Cell viability assessment by CCK-8 assay showing that MAOA overexpression significantly reduced the protective effect of MSC/NRG1β-Exo on OGD-injured neuronal cells compared to vector control. **(C)** Flow cytometry analysis of apoptosis using Annexin V/PI staining. **(D)** DCFH-DA fluorescence staining (green) for reactive oxygen species (ROS) detection with DAPI (blue) counterstaining. Scale bar = 50 μ m. **(E)** MAOA enzymatic activity was significantly increased in the MAOA group compared with vector control. **(F)** Quantification of oxidative stress markers in OGD-injured cells treated with MSC/NRG1β-Exo. **(G)** ELISA analysis of inflammatory cytokines in cell culture supernatants. All *in vitro* data represent *n* = 3 independent biological experiments. **p < 0.01 vs. Vector.

To further validate the functional significance of the miR-296-3p/MAOA axis in mediating the neuroprotective effects of MSC/NRG1β-Exo, we employed a gain-of-function approach by overexpressing MAOA in HT-22 cells. This complementary strategy allowed us to determine whether artificially increasing MAOA levels could override the protective effects of MSC/NRG1β-Exo, thereby establishing MAOA as a critical downstream effector in this pathway. Western blot analysis confirmed successful overexpression of MAOA in transfected cells compared to vector controls (Figure 6A). Importantly, MAOA overexpression significantly altered the expression of apoptosis-related proteins, reducing levels of the anti-apoptotic protein Bcl-2 while increasing the pro-apoptotic protein Bax, despite the presence of MSC/NRG1β-Exo. This shift in the Bcl-2/Bax ratio toward a pro-apoptotic state suggested that elevated MAOA levels could overcome the anti-apoptotic effects of exosome treatment. Consistent with this observation, cell viability assays revealed that MAOA overexpression significantly attenuated the protective effects of MSC/NRG1β-Exo under OGD conditions (Figure 6B). While cells transfected with the empty vector maintained high viability when treated with MSC/NRG1β-Exo, those overexpressing MAOA exhibited markedly reduced survival despite exosome treatment. This finding was further corroborated by flow cytometry analysis, which demonstrated that MAOA overexpression more than doubled the proportion of apoptotic cells even in the presence of MSC/NRG1β-Exo (Figure 6C). Since oxidative stress is a major mechanism through which MAOA mediates neuronal injury, we examined whether MAOA overexpression affected ROS production in our model. Intracellular ROS detection using the fluorescent probe DCFH-DA revealed substantially increased fluorescence intensity in MAOA-overexpressing cells compared to vector controls, indicating elevated oxidative stress despite MSC/NRG1β-Exo treatment (Figure 6D). Measurement of MAOA enzymatic activity confirmed enhanced catalytic function after MAOA overexpression (Figure 6E). This observation was further supported by biochemical analyses showing that MAOA overexpression significantly increased MDA levels (a marker of lipid peroxidation) while concurrently reducing SOD activity (an indicator of antioxidant capacity) (Figure 6F). Given the close relationship between oxidative stress and inflammation in ischemic injury, we also evaluated the impact of MAOA overexpression on inflammatory cytokine production. ELISA analysis of culture supernatants demonstrated that MAOA overexpression significantly elevated the levels of pro-inflammatory cytokines IL-1β, TNF-α, and IL-6 compared to vector controls, despite the presence of MSC/NRG1β-Exo (Figure 6G). This finding indicates that MAOA can override the anti-inflammatory effects of exosome treatment, further supporting its role as a key downstream mediator.

### Silencing of MAOA alleviates OGD-induced neuronal injury

**FIGURE 7:**
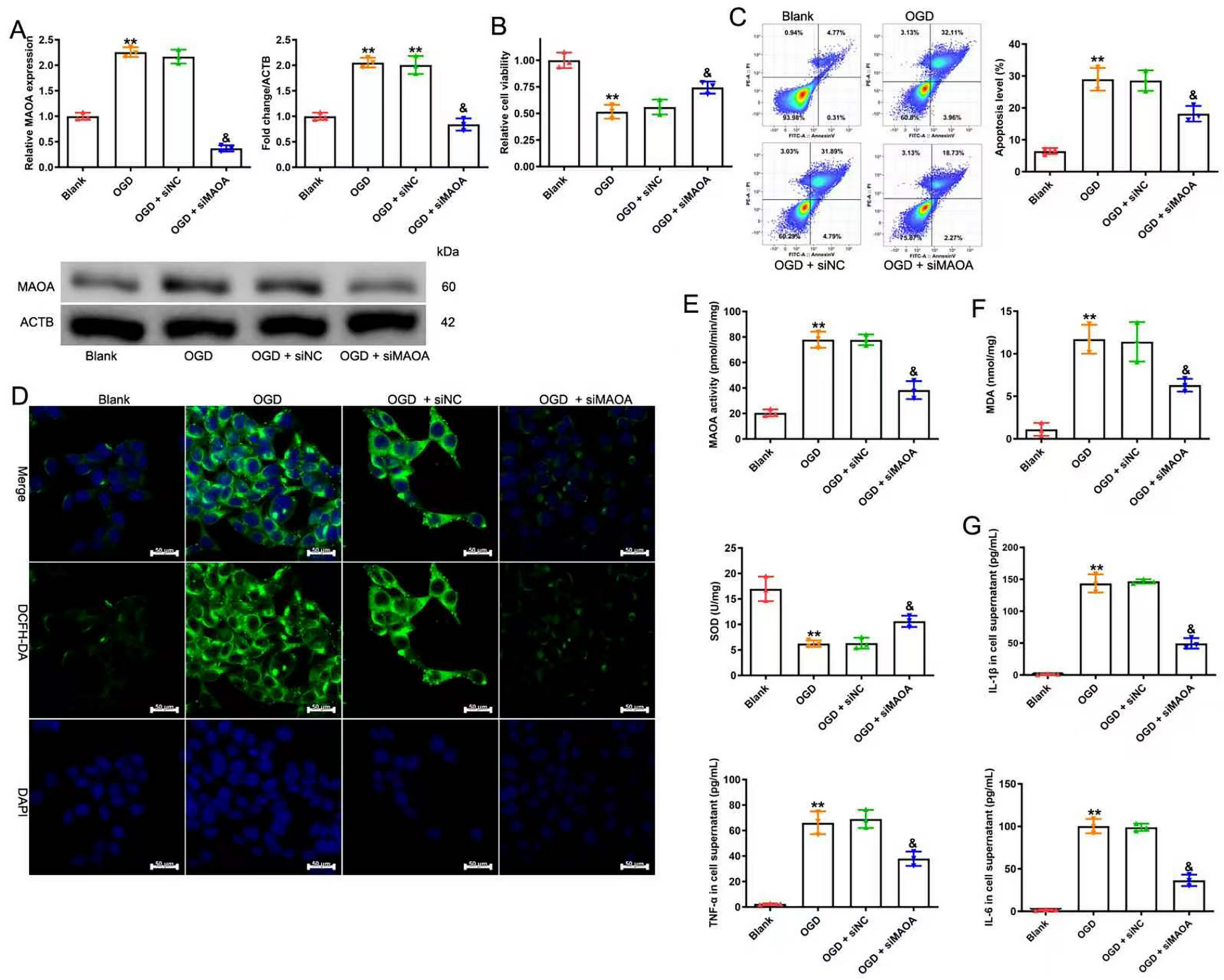
Monoamine oxidase A (MAOA) knockdown attenuates OGD-induced neuronal injury and reduces oxidative stress and inflammation. **(A)** qRT-PCR and Western blot analysis of MAOA expression in HT-22 cells under different conditions. OGD markedly upregulated MAOA, whereas siRNA-mediated knockdown (siMAOA) significantly reduced its expression compared with the negative control (siNC). **(B)** CCK-8 assay showing that MAOA silencing restored cell viability under OGD conditions. **(C)** Flow cytometry analysis of Annexin V/PI staining demonstrating that siMAOA significantly reduced OGD-induced apoptosis. Quantitative analysis is shown on the right. **(D)** Representative DCFH-DA fluorescence images (green) showing intracellular ROS levels. OGD increased ROS production, which was reduced by MAOA knockdown. Nuclei were counterstained with DAPI (blue). Scale bar = 50 μ m. **(E)** MAOA enzymatic activity was elevated after OGD and significantly suppressed by siMAOA. **(F)** Biochemical assays showing that siMAOA decreased malondialdehyde (MDA) levels and restored superoxide dismutase (SOD) activity. **(G)** ELISA quantification of pro-inflammatory cytokines IL-1β, TNF-α, and IL-6 in cell supernatants. MAOA silencing significantly reduced the OGD-induced increase in these inflammatory mediators. All *in vitro* data represent *n* = 3 independent biological experiments. **p < 0.01 vs. Blank; &*p* < 0.05 vs. OGD + siNC.

To further confirm the functional role of MAOA in OGD-induced neuronal injury, we performed loss-of-function experiments using siRNA-mediated knockdown. qRT-PCR and Western blot analysis revealed that OGD markedly increased MAOA expression in HT-22 cells, whereas siMAOA significantly reduced its expression compared with the negative control (siNC) (Figure 7A). Functionally, CCK-8 assays demonstrated that MAOA silencing restored cell viability under OGD conditions (Figure 7B). Consistent with this finding, flow cytometry analysis showed that siMAOA markedly decreased the proportion of apoptotic cells compared with OGD and OGD + siNC groups (Figure 7C). In addition, ROS detection using DCFH-DA fluorescence revealed that MAOA knockdown effectively suppressed OGD-induced oxidative stress (Figure 7D). Measurement of MAOA enzymatic activity further confirmed that siMAOA significantly reduced its catalytic function (Figure 7E). Biochemical assays showed that MAOA silencing decreased malondialdehyde (MDA) levels and concomitantly restored superoxide dismutase (SOD) activity (Figure 7F). Moreover, ELISA analysis of inflammatory cytokines demonstrated that siMAOA reduced the secretion of IL-1β, TNF-α, and IL-6 in OGD-treated cells (Figure 7G). Together, these results indicate that silencing MAOA effectively alleviates OGD-induced apoptosis, oxidative stress, and inflammation, further validating MAOA as a critical downstream effector in ischemic neuronal injury.

## Discussion

Cerebral ischemic stroke remains a serious health issue with limited treatment options (Hu et al., 2017; Sarraj et al., 2024). In the present study, we demonstrated that exosomes derived from NRG1β-overexpressing mesenchymal stem cells exerted marked neuroprotective effects in both MCAO and OGD models. Compared with conventional MSC-derived exosomes, MSC/NRG1β-Exo more effectively reduced infarct volume, alleviated neuronal apoptosis, suppressed oxidative stress and inflammatory responses, and improved neuronal survival. Mechanistically, these protective effects were mediated, at least in part, through the miR-296-3p/MAOA axis.

We first confirmed the identity and purity of mesenchymal stem cells (MSCs) and their exosomes in accordance with established standards (Théry et al., 2018). Consistent with prior studies (Wang J. et al., 2020; Xin et al., 2013), our study confirmed that unmodified MSC-derived exosomes already exerted protective effects against cerebral ischemic damage. More importantly, NRG1β modification significantly enhanced this therapeutic efficacy, indicating that genetic engineering of MSCs is an effective strategy to optimize exosome-based therapy. Given the well-established neuroprotective role of NRG1β in neuronal survival, anti-apoptosis, and anti-inflammatory regulation, our findings further extend previous work by showing that NRG1β can potentiate the bioactivity of MSC-derived exosomes rather than being delivered as a free protein alone.

A key finding of this study is the identification of miR-296-3p as an important effector molecule enriched in MSC/NRG1β-Exo. Among the candidate miRNAs identified, miR-296-3p showed the most significant upregulation in NRG1β-MSC-derived exosomes. Inhibition of miR-296-3p abolished the protective benefits of these exosomes, while bioinformatic analysis and experimental validation-including dual-luciferase reporter assays and RNA immunoprecipitation-confirmed that miR-296-3p directly targets the 3′UTR of monoamine oxidase A (MAOA). MAOA, a mitochondrial enzyme linked to oxidative stress and neuronal damage (Georgieva et al., 2024; van Rhijn et al., 2022), when overexpressed, reversed the beneficial effects of MSC/NRG1β-Exo, underscoring the critical role of the miR-296-3p/ MAOA axis.

The miR-296-3p/MAOA axis provides a compelling explanation for the neuroprotective effects of MSC/NRG1β-Exo. Under ischemic conditions, MAOA degrades monoamine neurotransmitters, producing hydrogen peroxide as a byproduct, which intensifies oxidative stress and mitochondrial dysfunction (Dorfman et al., 2014). By downregulating MAOA expression through targeting its 3′UTR, miR-296-3p mitigates oxidative stress, as demonstrated by reduced MDA levels and elevated SOD activity in our models. This alleviation of oxidative burden further contributed to decreased apoptosis-reflected in an improved Bcl-2/Bax ratio-and suppressed inflammation, evidenced by reduced levels of IL-1β, TNF-α, and IL-6. Compared to conventional MSC-derived exosomes, MSC/NRG1β-Exo enhanced the delivery of miR-296-3p, likely through NRG1β-mediated transcriptional regulation, thereby amplifying therapeutic outcomes. While the neuroprotective role of NRG1β has been documented previously (Li et al., 2007; Noll et al., 2019), integrating it into exosomes and unveiling the specific mechanism of the miR-296-3p/MAOA axis represents a critical advancement that sets our study apart from earlier work.

Despite these promising findings, several limitations of our study should be acknowledged. First, our *in vivo* experiments mainly focused on the acute phase after cerebral ischemia, and longer observation periods are needed to determine whether MSC/NRG1β-Exo can improve long-term neurological function and tissue remodeling. Second, although MAOA was validated as a direct target of miR-296-3p, this microRNA may regulate additional genes involved in neuroprotection, which were not explored here. Third, while our findings demonstrate promising preclinical efficacy, issues such as optimal dose, delivery route, pharmacokinetics, large-scale production, and biosafety of engineered exosomes still require further investigation before clinical translation.

## Conclusion

In conclusion, our study demonstrates that exosomes derived from NRG1β-overexpressing MSCs provide significant neuroprotection in ischemic conditions through a novel miR-296-3p/MAOA regulatory axis. Mechanistically, this effect is mediated, at least in part, through enrichment of miR-296-3p, which directly suppresses MAOA and thereby attenuates oxidative stress, inflammation, and apoptosis. These findings not only deepen our understanding of the molecular basis of engineered exosome therapy, but also suggest that the miR-296-3p/MAOA axis may represent a promising therapeutic target for ischemic stroke.

## Data Availability

The raw data supporting the conclusions of this article will be made available by the authors, without undue reservation.
